# Comparing the visual outcome, visual quality, and satisfaction among three types of multi-focal intraocular lenses

**DOI:** 10.1038/s41598-020-69318-y

**Published:** 2020-09-09

**Authors:** Dong Won Paik, Jun Sang Park, Chan Min Yang, Dong Hui Lim, Tae-Young Chung

**Affiliations:** 1 Department of Ophthalmology, Sungkyunkwan University School of Medicine, Samsung Medical Center, Seoul, Republic of Korea; 2 Department of Medicine, The Graduate School, Sungkyunkwan University, Seoul, Republic of Korea; 3 Nune Eye Hospital, Seoul, Republic of Korea; 4 Department of Medical Device Management and Research, Samsung Advanced Institute for Health Sciences and Technology, Sungkyunkwan University, Seoul, Republic of Korea

**Keywords:** Quality of life, Lens diseases, Vision disorders, Eye manifestations

## Abstract

This study compared the visual outcome, visual quality, and satisfaction following implantation of the Mix-and-Match bifocal IOLs (+ 2.75 D and + 3.25 D add power Tecnis Multifocal Model), EDOF IOL (Tecnis Symfony IOL), and Trifocal IOL (FineVision PodFT, PhysIOL). All outcomes were compared among the three groups. The manifest refraction indicated that the EDOF group had significantly higher myopic spherical equivalent values than did the others. In the terms of visual acuity, there were no significant differences in far or intermediate visual acuity among the three groups. Only in near (33 cm), the EDOF group had significantly worse binocular visual acuity than did the Trifocal group (*p* = 0.002). Regarding to defocus curve, the Trifocal group had better defocus curves at near distances (− 2.0 to − 3.5 D; *p* = 0.001 vs. EDOF) than did the other two groups. In contrast sensitivity test, the EDOF group had relatively lower value than did the other two groups. In reading speed, only at 0.3 logMAR (6.5-point font), Mix-and-Match group had a significantly higher reading speed than did the other two groups (*p* =  < 0.001 vs. EDOF, *p* = 0.007 vs. Trifocal). also Mix-and-Match group showed significantly fewer visual artifacts. There were no differences between the three groups in terms of patient satisfaction.

**ClinicalTrials.gov number**: NCT04019691.

## Introduction

The monofocal lens, which was primarily used for cataract surgery in the past, was applied to one target (e.g., near or far). Therefore, depending on the target, long-distance glasses or reading glasses were essential after cataract surgery. More recently, patients ask for simultaneous correction of cataracts and presbyopia. Therefore, medical technology is evolving according to this demand. Efforts are now being made to correct intermediate visual acuity, as well as far and near distance at the same time. Consequently, various multifocal intraocular lenses (IOLs) have been developed. Initially, the diffractive bifocal IOL was most widely used^1^.

Diffractive bifocal IOLs provide somewhat poor intermediate visual acuity^2^. However, modern society increases the need for intermediate visual acuity, such using a computer or doing outdoor activities. These societal changes have led to an increase in patient demand for improved intermediate visual acuity.

The Mix-and-Match method was introduced to increase intermediate visual acuity^3,4^. In this method, bifocal IOLs with different add powers are implanted. Recently, an extended depth of focus (EDOF) IOL, and a Trifocal IOL (that combines two types of bifocal IOLs in one lens) have been developed^5,6^.

Studies comparing two of the above three methods have been published^7,8^. However, no prior studies have compared all three methods at the same time. Therefore, the purpose of this study was to compare these three methods for improving intermediate visual acuity for the first time.

Here we compared visual outcome, visual quality, and patient satisfaction among bilateral implantation of the Mix-and-Match bifocal IOLs (+ 2.75 D and + 3.25 D add power Tecnis Multifocal Model), EDOF IOL (Tecnis Symfony IOL), and Trifocal IOL (FineVision PodFT, PhysIOL).

## Results

Each of the three groups (Mix-and-Match, EDOF and Trifocal group) included 20 patients (40 eyes). There were no significant differences in age, sex, spherical diopter, cylinder diopter, or spherical equivalent (SE) between the three groups preoperatively (Table 1). Although there was no significant difference in the preoperative uncorrected visual acuity between the three groups, the Mix-and-Match group had significantly better preoperative best-corrected visual acuity than did the other groups (*p* = 0.004) (Table 1).

**Table 1:** Patient demographics.

	Mix and Match	EDOF	Trifocal	*p* value
Patients (*n*)	20	20	20	
Age (years)	60.13 ± 6.52	60.44 ± 9.72	56.78 ± 6.75	0.324
Sex (M:F)	8:12	7:13	6:14	0.472
Sph (D)	− 1.26 ± 3.84	− 0.61 ± 2.53	− 1.97 ± 3.67	0.23
Cyl (D)	− 0.76 ± 0.51	− 0.89 ± 0.55	− 0.89 ± 0.63	0.548
Spherical equivalent (D)	− 1.65 ± 3.81	− 1.06 ± 2.55	− 2.41 ± 3.67	0.228
UCVA (logMAR)	0.58 ± 0.45	0.49 ± 0.35	0.63 ± 0.50	0.371
BCVA (logMAR)	0.15 ± 0.20	0.35 ± 0.28	0.24 ± 0.28	0.004

*UCVA* uncorrected visual acuity, *BCVA* best corrected visual acuity, *logMAR* logarithm of the minimal angle of resolution, *Sph* sphere diopter, *Cyl* cylinder diopter.

**Figure 1:**
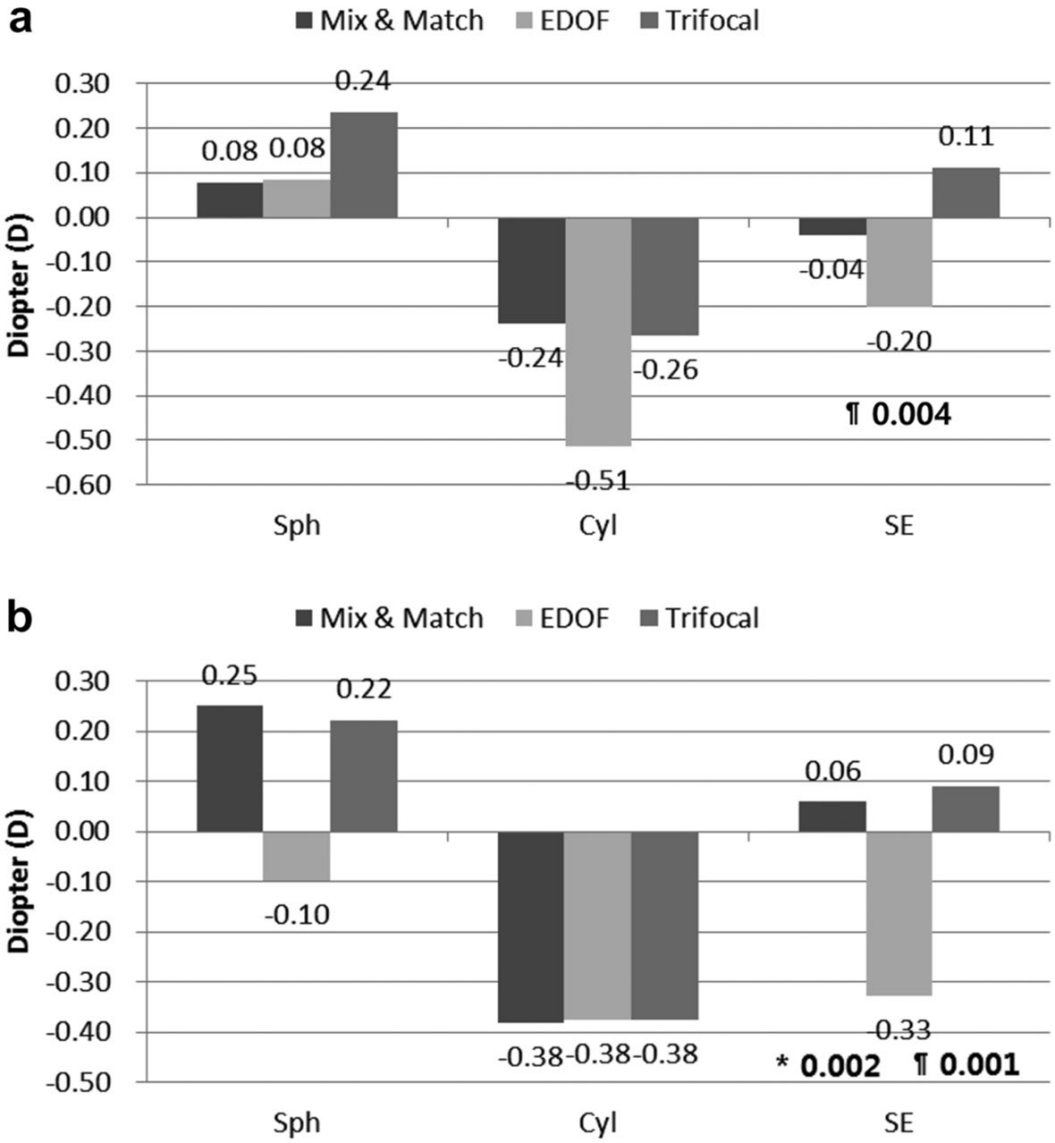
Postoperative refractive outcomes in the three groups (Mix-and-Match, EDOF and Trifocal). **a** Dominant eye. The EDOF group had significantly higher myopic SE values than did the Trifocal group (*p* = 0.004) in the dominant eyes at three months. **b** Non-dominant eye. The EDOF group had higher myopic SE values than did the Mix-and-Match group (*p* = 0.002) and the Trifocal group (*p* = 0.001) in non-dominant eyes at three months. All outcomes were compared among the three groups. Bonferroni correction for multiple comparisons: significant *p* values (*p* < 0.017) in bold with symbols. *: Mix and Match versus EDOF, §: Mix and Match versus Trifocal, ¶: EDOF versus Trifocal.

The manifest refraction performed three months postoperatively indicated that the EDOF group had significantly higher myopic SE values than did the Trifocal group (*p* = 0.004) in the dominant eyes. In subgroup analysis, the EDOF group also had higher myopic SE values than did the Mix-and-Match (*p* = 0.002) and Trifocal groups (*p* = 0.001) in the non-dominant eyes (Fig. 1).

**Figure 2:**
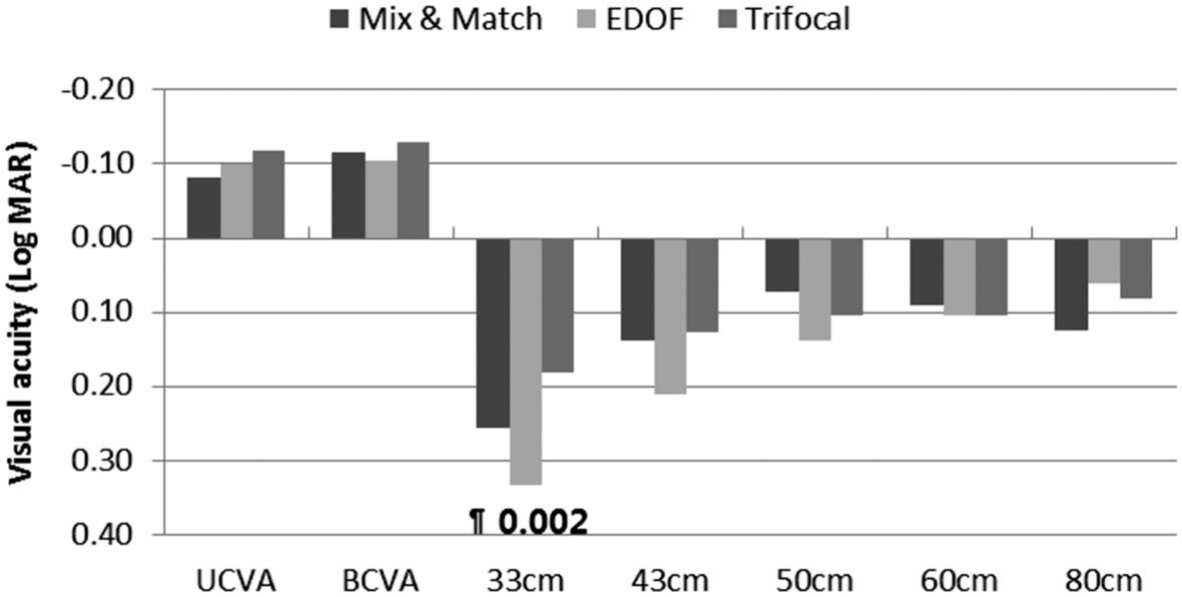
Postoperative uncorrected near, intermediate, and far visual acuity. No significant differences in far or intermediate visual acuity among the three groups were observed in the binocular vision test at three months. In subgroup analysis, at 33 cm near distance, the EDOF group had significantly worse binocular visual acuity than did the Trifocal group (*p* = 0.002). All outcomes were compared among the three groups. Bonferroni correction for multiple comparisons: significant *p* values (*p* < 0.017) in bold with symbols. *: Mix and Match versus EDOF, §: Mix and Match versus Trifocal, ¶: EDOF versus Trifocal.

There were no significant differences in far or intermediate visual acuity among the three groups were observed in the binocular vision test performed at three months postoperatively. However, in the subgroup analysis, the EDOF group had significantly worse binocular visual acuity than did the Trifocal group at 33 cm near distance (*p* = 0.002), and dominant eye also showed similar results (Table2) (Fig. 2, Fig A1).

**Table 2:** Binocular visual acuities at preoperative 3 months after surgery (logMAR).

	Mix and Match	EDOF	Trifocal	*p* value
UCVA at 5 m	− 0.08 ± 0.10	− 0.10 ± 0.07	− 0.12 ± 0.08	0.411
BCVA at 5 m	− 0.12 ± 0.08	− 0.10 ± 0.06	− 0.13 ± 0.08	0.608
UIVA at 80 cm	0.12 ± 0.14	0.06 ± 0.08	0.08 ± 0.10	0.212
UIVA at 60 cm	0.09 ± 0.09	0.10 ± 0.12	0.10 ± 0.10	0.887
UNVA at 50 cm	0.07 ± 0.11	0.14 ± 0.11	0.10 ± 0.11	0.213
UNVA at 43 cm	0.14 ± 0.09	0.21 ± 0.14	0.13 ± 0.12	0.084
UNVA at 33 cm	0.25 ± 0.11	0.33 ± 0.14	0.18 ± 0.10	0.003

*UCVA* uncorrected visual acuity, *BCVA* best corrected visual acuity, *UIVA* uncorrected intermediate visual acuity, *UNVA* uncorrected near visual acuity, *logMAR* logarithm of the minimal angle of resolution.

**Figure 3:**
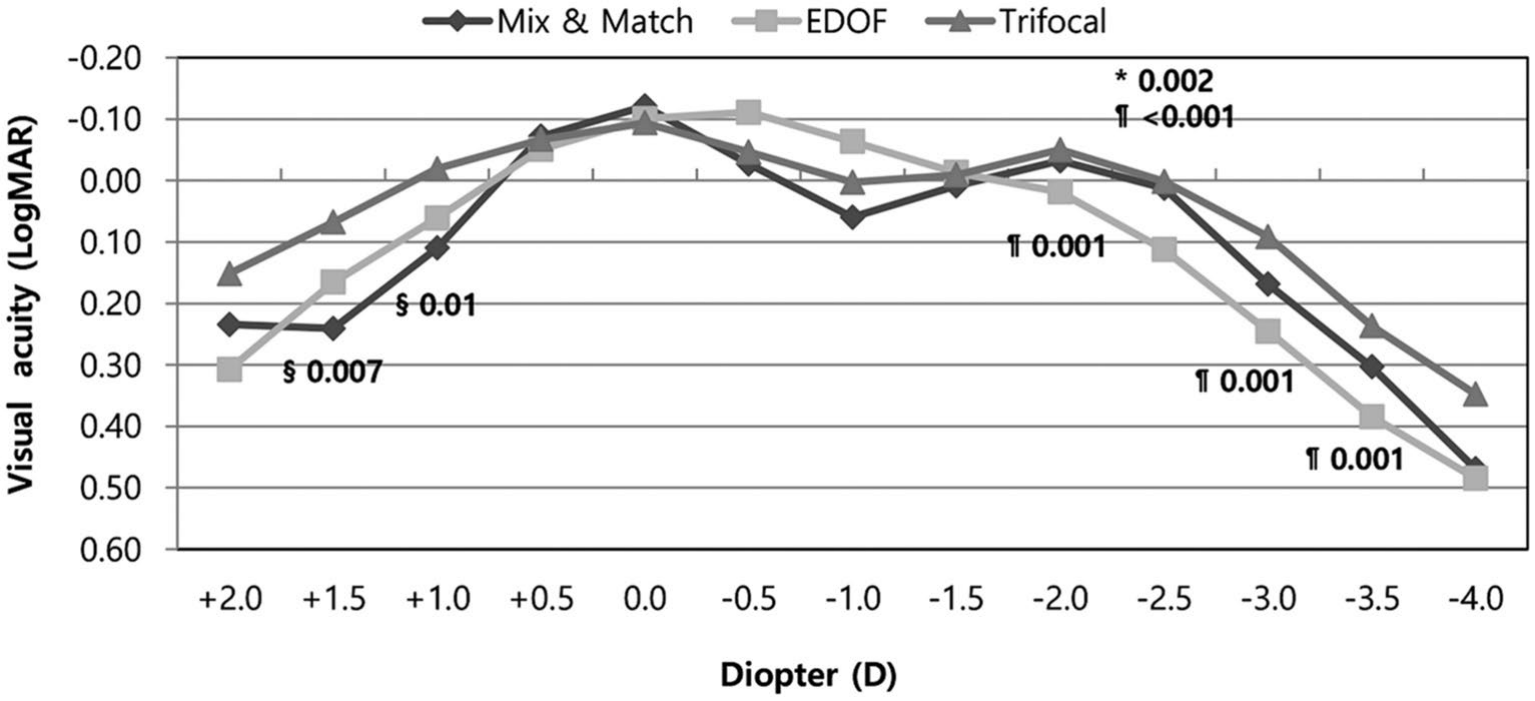
Binocular defocus curves for the three groups. Mix-and-Match and Trifocal groups showed best visual acuity results at 0.0 D and second peak at − 2.0 D, but showed dips in the intermediate range (− 0.5 to − 1.5 D). The EDOF group provided continuous range of vision, but with a more progressive visual acuity decrease to the higher levels of defocus. Trifocal group had a significantly better defocus curve at near distance (− 2.0 to − 3.5 D; *p* = 0.001 vs. EDOF) than did the EDOF group in the subgroup analysis. All outcomes were compared among the three groups. Bonferroni correction for multiple comparisons: significant *p *values (*p* < 0.017) in bold with symbols. *: Mix and Match versus EDOF, §: Mix and Match versus Trifocal, ¶: EDOF versus Trifocal.

In the defocus curve tests, the Mix-and-Match and Trifocal groups showed best visual acuity results at 0.0 D and at-2.0 D. However, these groups showed dips in visual acuity in the intermediate range (− 0.5 to − 1.5 D).In contrast, the EDOF group demonstrated a smoother profile along the whole defocus curve. The EDOF group showed a more progressive visual acuity decrease at the higher levels of defocus. The Trifocal group had better defocus curves at near distances (− 2.0 to − 3.5 D; *p* = 0.001 vs. EDOF) than did the other two groups. The Trifocal group also had significantly better defocus curves than did the EDOF group in the subgroup analysis. From the point of view of ‘depth of focus’, which defined as the range of lens power (from zero defocus to the largest negative power) over which the mean acuity is 0.2 logMAR (20/32) or better, Mix-and-Match and Trifocal group (0 to − 3D) showed longer depth of focus than the EDOF group (0 to − 2.5D) in binocular defocus curves (Fig. 3). In monocular defocus curves, Trifocal group had the longest range of defocus from 0 to − 3.0 D (VA above 0.2 logMAR) in both dominant eye and non-dominant eye (Fig A2).

**Figure 4:**
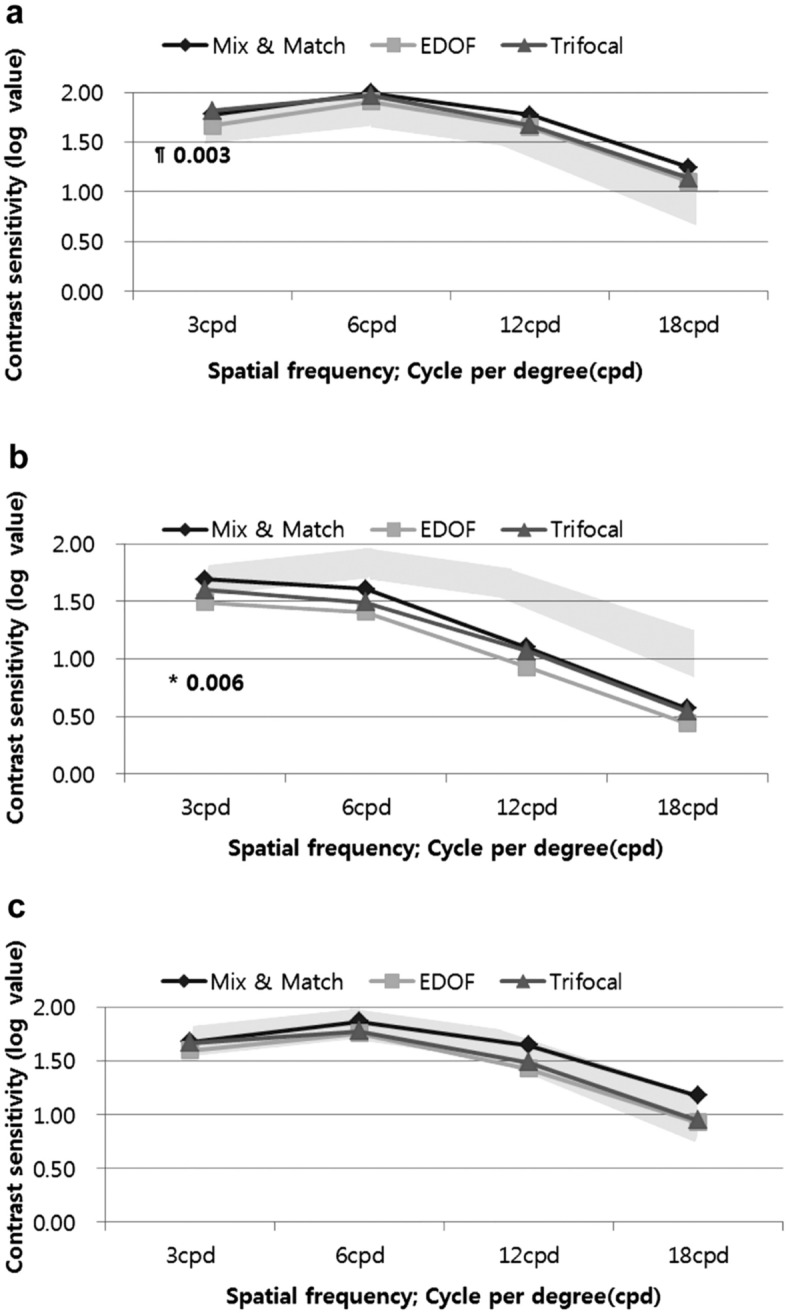
Contrast sensitivity in the three groups. **a** Photopic. At the spatial frequency of 3 cycles per degree (cpd), the EDOF group was significantly lower than the Trifocal group under the photopic condition at three months (*p* = 0.003). **b** Mesopic with glare off. At the spatial frequency of 3 cycles per degree (cpd), the EDOF group was significantly lower than the Mix-and-Match group at three months (*p* = 0.006). **c** Mesopic with glare on. All three groups had normal values and no differences. All outcomes were compared among the three groups. Bonferroni correction for multiple comparisons: significant *p *values (*p* < 0.017) in bold with symbols. *: Mix and Match versus EDOF, §: Mix and Match versus Trifocal, ¶: EDOF versus Trifocal. Opaque area represents the normal value range.

All three groups had normal values under the photopic and mesopic conditions with glare conditions in the contrast sensitivity test. The EDOF group had relatively lower value of contrast sensitivity than did the other two groups. At the spatial frequency of 3 cycles per degree (cpd), the contrast sensitivity was significantly lower than that of the Trifocal group under the photopic condition (*p* = 0.003), and lower than that of the Mix-and-Match group under the mesopic condition (*p* = 0.006) (Fig. 4).

**Figure 5:**
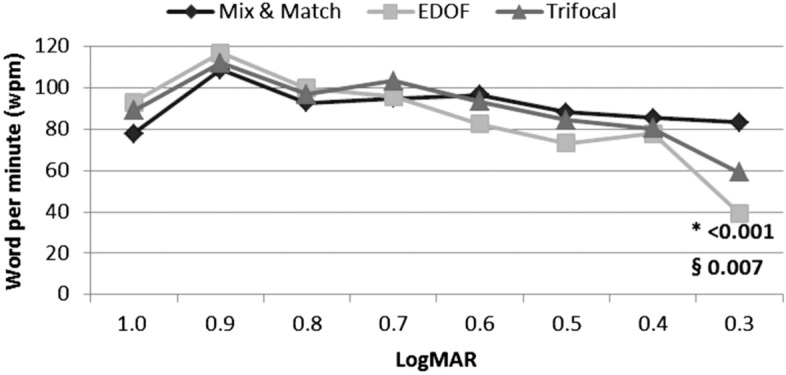
Reading speed test results for the three groups. No significant differences among the three groups for font sizes between 1.0 logMAR (29-point font) and 0.4 logMAR (9-point font). The Mix-and-Match group had a significantly higher reading speed than the other two groups at 0.3 logMAR (6.5-point font) (*p* =  < 0.001 vs. EDOF, *p* = 0.007 vs. Trifocal). All outcomes were compared among the three groups. Bonferroni correction for multiple comparisons: significant *p* values (*p* < 0.017) in bold with symbols. *: Mix and Match versus EDOF, §: Mix and Match versus Trifocal, ¶: EDOF versus Trifocal.

There were no significant differences among the three groups in reading speed for font sizes between 1.0 logMAR (29-point font) and 0.4 logMAR (9-point font). However, the Mix-and-Match group had a significantly higher reading speed than did the other two groups at 0.3 logMAR (6.5-point font) (*p* =  < 0.001 vs. EDOF, *p* = 0.007 vs. Trifocal) (Fig. 5). All patients with Mix-and-Match IOLs were able to read letters with the following sizes: 0.22 logMAR (5.5-point font), 0.1 logMAR (4.5-point font), and 0 logMAR (3.5-point font). In contrast, patients in the other two groups were unable to read letters in the above font sizes.

**Figure 6:**
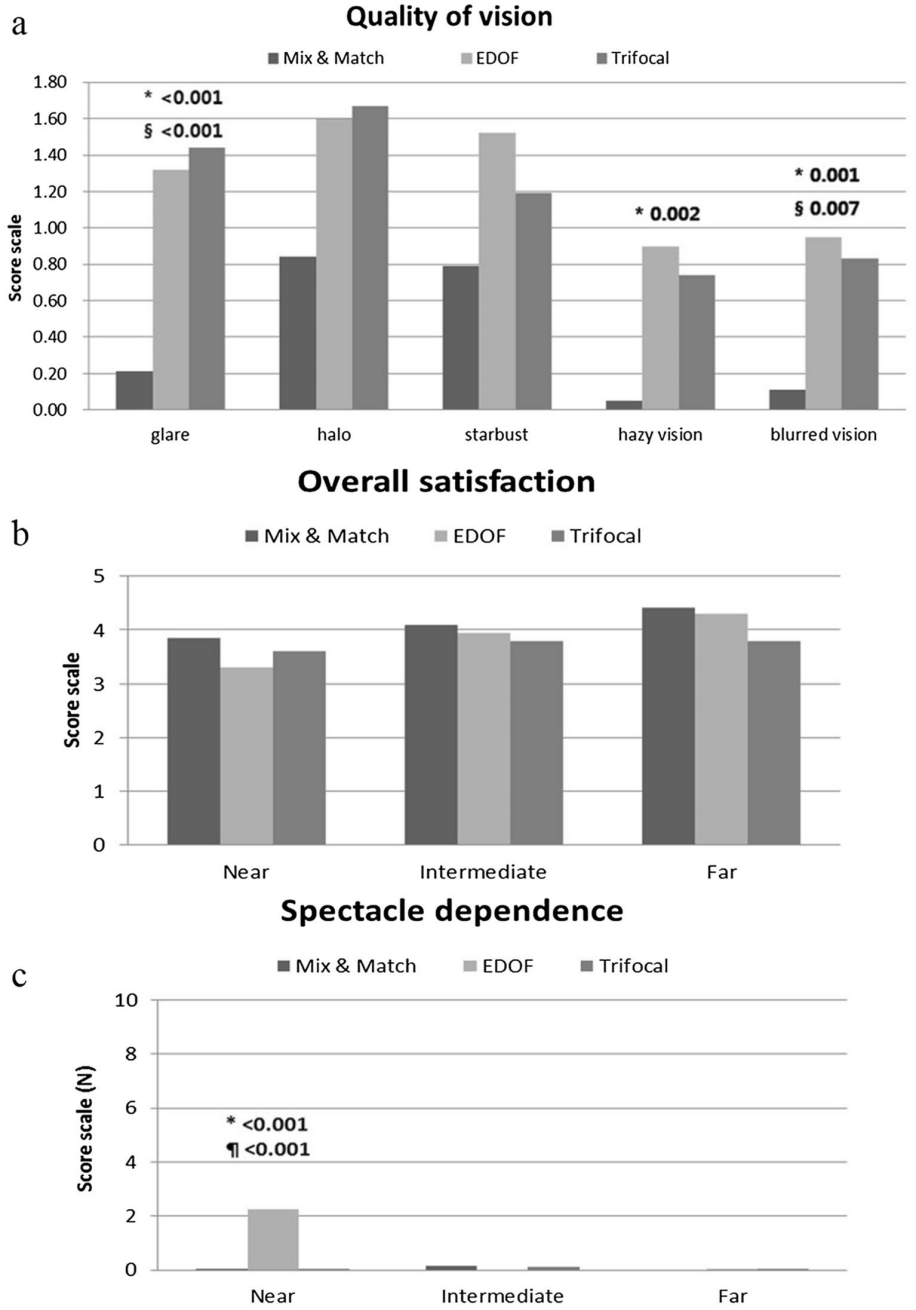
Postoperative questionnaire at 3 months. **a** Quality of vision (visual artifacts) questionnaire. The Mix-and-Match group showed the lowest frequency of visual artifacts at three months. Subgroup analysis showed the frequency of three symptoms (glare, hazy vision, and blurred vision) were significantly lower in the Mix-and-Match group than they were in the other groups (glare; *p* < 0.001 vs. EDOF and *p* < 0.001 vs. Trifocal, hazy vision; *p* = 0.002 vs. EDOF, blurred vision; *p* = 0.001 vs. EDOF and *p* = 0.007 vs. Trifocal). **b** Overall satisfaction (near, intermediate, and far) questionnaire. Three groups reported relatively similar satisfaction with far, intermediate, and near vision at three months. **c** Spectacle dependence (near, intermediate, and far) in the three groups. The EDOF group showed significantly higher near-vision spectacle dependence (*p* < 0.001 vs. Mix-and-Match, *p* < 0.001 vs. Trifocal) at 3 months. All outcomes were compared among the three groups. Bonferroni correction for multiple comparisons: significant *p *values (*p* < 0.017) in bold with symbols. Spectacle dependence score scale: 0–10 (0—none; N—N out of 10; 10—Always). Quality of vision score scale: 0–3 (0—none; 1—mild; 2—moderate; 3—severe). Subjective satisfaction score scale: 1–5 (1—very unsatisfied; 2—unsatisfied; 3—neither satisfied nor dissatisfied; 4—satisfied; 5—very satisfied). *: Mix and Match versus EDOF, §: Mix and Match versus Trifocal, ¶: EDOF versus Trifocal.

The Mix-and-Match group had the lowest frequency of visual artifacts among all three groups. Subgroup analysis showed the frequency of three symptoms (glare, hazy vision. and blurred vision) were significantly lower in the Mix-and-Match group than they were in the other groups (glare; *p* < 0.001 vs. EDOF and *p* < 0.001 vs. Trifocal, hazy vision; *p* = 0.002 vs. EDOF, blurred vision; *p* = 0.001 vs. EDOF and *p* = 0.007 vs. Trifocal) (Fig. 6a).

All three groups reported relatively similar satisfaction with far, intermediate, and near vision (Fig. 6b). A questionnaire that assessed postoperative spectacle dependence revealed a significantly higher near-vision spectacle dependence in the EDOF group compared to the other groups (*p* < 0.001 vs. Mix-and-Match, *p* < 0.001 vs. Trifocal) (Fig. 6c).

## Discussion

The existing diffractive bifocal IOLs have been shown to provide good near and far visual acuities, but not intermediate visual acuity. Various methods have been developed to overcome this limitation. In this study, the mix and match bifocal IOL, EDOF IOL, and trifocal IOL were implanted. We compared these three methods with regard to clinical outcomes and measurements of patient satisfaction were compared among the three groups. Each lens had its advantages and disadvantages. First, the Mix-and-Match group had poorer intermediate visual acuity, but better visual quality than compared to those of the other groups. Second, in the EDOF group, the visual changes from far to near vision occurred steadily; however, this group had poorer near visual acuity than did the other groups. Third, the Trifocal group had good far, intermediate, and near visual acuity. However, the Trifocal group also experienced relatively more glare and halo symptoms than did the other groups.

Recently studies have found that the Symfony IOL is associated with poor near visual acuity^8,9^. With these reports in mind, a slightly myopic target diopter was used for the EDOF group during cataract surgery. Therefore, the SE of the dominant and non-dominant eyes in the EDOF group were more myopic than those observed in the other groups. Choosing a slightly myopic one under the same conditions could remain a limitation of this study and lead to unintended bias. However, the EDOF group still had poorer near visual acuity than did the other groups. Furthermore, in subgroup analysis, a significant difference between the EDOF and Trifocal groups was observed in the near-distance interval (− 2.0  to − 3.5 D) on the defocus curve. Taken together, these results indicate that despite the use of a slightly myopic target diopter in the EDOF group, the lower near visual acuity was not overcome (when compared to that of the other groups).

The advantage of the EDOF IOL is that it does not show any dip phenomenon in the defocus curve. Instead, there was a gradual decrease from far to near distances with the EDOF IOL. Based on this observation, a previous study reported that the Symfony IOL provides better intermediate visual acuity than do the existing diffractive bifocal IOLs^10^. In this study, the EDOF group had relatively better intermediate visual acuity on the defocus curve than did the Mix-and-Match group. Therefore, the trifocal IOL is the best among the three for near distances. The mix and match method followed Trifocal IOL for near distances. The EDOF IOL was the best for intermediate distances among the three groups, followed by the Trifocal IOL.

Previous studies have found that mix-and-match bi-focal IOLs with different add powers improved intermediate visual acuity^3,4^. However, we found that even with the mix-and-match method, the intermediate visual acuity in the defocus curve was worse than that obtained using Symfony IOLs. Unfortunately, no prior studies have directly compared the visual acuity of mix-and-match bi-focal IOLs and Symfony IOLs. Therefore, there is a need for further studies comparing the intermediate distance visual acuity of the two methods. Similar to our study, other reports have also found no significant difference in the intermediate visual acuity between the trifocal IOLs and Symfony IOLs^8,9,11^.

In the contrast sensitivity test performed in this study, the Mix-and-Match group had relatively better results than did the other groups in most intervals. The bi-focal IOL generally led to a higher contrast sensitivity than did the trifocal IOL. This result may be due to light dissipation, which is a characteristic phenomenon of diffractive IOLs that causes the formation of three foci and leads to a relative decrease in light brightness. A recent study by Mojziset al. reported that contrast sensitivity was higher with bifocal IOLs than it was with trifocal IOLs^12^.

In a study comparing contrast sensitivity among four lenses[including monofocal IOLs, two different near add power bifocal IOLs (ReSTOR + 2.5 D IOL or ReSTOR + 3.0 D IOL), and the Symfony IOLs], patients with the monofocal and Symfony IOLs showed similar contrast sensitivity values, both of which were significantly higher than those reported by patients using the bifocal IOLs^10^. The results of the above study are inconsistent with our findings that the EDOF group had an overall lower contrast sensitivity than did the Mix-and-Match group. Unfortunately, there is a lack of studies comparing the contrast sensitivity of bifocal IOLs and Symfony IOLs. Therefore, a comparative analysis is difficult.

Although the Mix-and-Match group had relatively better contrast sensitivity values at most intervals in our study, there were no large differences compared to those of the other two groups. The important point is that all three groups had normal values under the photopic and mesopic conditions with glare conditions; therefore, these differences were clinically insignificant. As a result, none of the three methods are predicted to not interfere with patient daily life after cataract surgery.

Good near visual acuity does not always indicate good functional near vision, which is necessary for reading books or newspapers. Therefore, recent studies have used a reading speed test, which reflects functional near vision, to determine the clinical outcome of multifocal IOLs^13,14^.

One study that compared the reading speeds of patients with a bifocal IOL (AT Lisa,) to those of patients with Trifocal IOL(AT Lisa Tri)found no significant difference between the two groups^14^. Another study reported no difference in reading speed between patients with a bifocal IOL (ReSTOR) and those with the Symfony IOL^13^. Similar results were obtained in this study. There were no significant differences in the reading speed for most font sizes among the three groups. However, for very small font sizes, the Mix-and-Match group had faster reading speeds than did the other groups. It is possible that differences in the testing method and language characteristics contribute to variation in the results^14,15^.

In the questionnaire of quality of vision conducted in this study, the Mix-and-Match group showed the best results, while the EDOF and Trifocal groups had comparable results. Trifocal IOLs are required to split more light energy to form the third focal point than are bifocal IOLs. This fact may have an effect on the vision quality in near and far distances. Gundersen and Potvin have published a study comparing the quality of vision between patients with mix-and-match bifocal IOLs and those with trifocal IOLs^16^. The group found that patients with trifocal IOLs complained of more discomfort due to visual artifacts than did those with bifocal IOLs. However, several other studies have compared found no difference in the quality of vision between patients with the Symfony IOL and those with trifocal IOLs, and have reported no difference between the two groups^9,11^. The results of our study were similar to those of previous studies that compared the visual quality among patients with multifocal IOLs.

There was no significant difference in the three groups with regard to overall satisfaction in the self-report questionnaire for near, intermediate, or far vision. This result was inconsistent with the results of the visual acuity and defocus curve tests. These differences may be due to the differences between the subjective questionnaire and the objective tests.

In the questionnaire survey of postoperative spectacle dependence, only patients in the EDOF group had a high reliance on near-vision spectacles. This finding is consistent with the result of the objective test conducted in this study. It is clear that near vision was insufficient in the EDOF group, compared with that in the other groups.

This study has some limitations. First, this study used a non-randomized method to compare multifocal IOLs. Although this is certainly commendable from a clinical and ethical point of view, it may lead to selection bias. Second, not all IOL selections aimed at emmetropia. According to previous studies, there are several ways to select the appropriate EDOF IOL. In the case of binocular injection of EDOF IOL, both eyes may be aiming for emmetropia, or the non-dominant eye may be targeted for micro-monovision with a slightly myopic target^17^. However, in this study, EDOF IOL was selected by the micro-monovision method. Basically in dominant eyes, we aimed for emmetropia, but when necessary to select, we chose among the myopic targets closest to emmetropia. And non-dominant eyes with slightly myopic target. So this might also induce unwanted bias. Third, subjective measures of photic phenomena and quality of vision only may not be sufficient to compare multifocal IOLs of different features. Additional research using objective assessment methods may also be necessary. At last, although our study is limited by its small sample size, it has two major advantages. Our prospective study is the first to simultaneously compare three types of IOLs including the mix-and-match method using bifocal IOLs. We also comprehensively evaluated clinical outcomes, including reading performance, contrast sensitivity, and other subjective measures, which were assessed using a questionnaire.

In conclusion, each of the three methods studied has both advantages and disadvantages. The Mix-and-Match group had the best visual quality among the three groups, as well as the fastest reading speed with a small font size. However, in the defocus curve tests, the Mix-and-Match group exhibited a severe reduction in the intermediate distance range. Therefore, the intermediate-distance visual acuity of defocus curve in the Mix-and-Match group was the lowest among the three groups. The EDOF group had the most stable visual acuity without an abrupt visual deterioration in the entire defocus curve test. Therefore, the best intermediate-distance visual acuity of the defocus curve was observed in this group. However, near visual acuity of the vision test and the defocus curve was lowest in the EDOF group. Therefore, dependence on near-vision spectacles was high after surgery in the EDOF group. The Trifocal group had good visual acuity at near, intermediate, and far distances. However, the Trifocal group also had the most disadvantages in the quality of vision questionnaire. Since the life circumstances of people who undergoing cataract surgery vary from person to person; therefore, the ideal IOL would optimize postoperative quality of life. We believe that our study will be helpful in choosing a suitable multifocal IOL for cataract surgery.

## Materials and methods

### Subjects

This prospective study analyzed 120 eyes from 60 patients who underwent bilateral cataract surgery (performed by a single surgeon) at the presenter institution. The patients were divided into three groups, with each group comprising 20 patients and 40 eyes. All patients were assessed for principle vision requirements in terms of their near-, intermediate-, and distance-vision. The choice of IOL proposed for implantation was based on their responses^10^. The patients in the Mix-and-Match group underwent implantation of a TecnisZKB00 multifocal IOL with + 2.75 D add power (Abbott Medical Optic Inc., Santa Ana, CA) in the dominant eye, and a Tecnis ZLB00 multifocal IOL with + 3.25 D add power (Abbott Medical Optics Inc.) in the non-dominant eye. In patients in the EDOF group, the Tecnis Symfony ZXR00 trifocal IOLs (Abbot Medical Optics Inc.) were implanted in both eyes. In patients in the Trifocal group, FineVisionPodFT IOLs (PhysIOL SA) were implanted in both eyes.

The study was approved by the Institutional Review Board of Samsung Medical Center. This study adhered to the tenets of the Declaration of Helsinki. Before participating in the study, written informed consent was obtained from all patients. This study was also registered at clinicaltrials.gov (NCT04019691) and the date of registration was July 15, 2019.

Patients were included if they were diagnosed with bilateral senile cataracts and the desire to be spectacle-free for all distances. Patients were excluded if they were younger than 21 years, had corneal astigmatism > 1.00 D, previous ocular surgery or trauma, or ocular disease other than cataracts. The hole-in-the-card test was performed in all patients for detection of the dominant eye preoperatively.

Preoperatively, the axial length, corneal refractive power, and chamber depth were measured using IOL Master version 5.4 (Carl Zeiss Meditec, Jena, Germany) to determine the IOL refractive power. The target diopter was determined by inputting the axial length, corneal refractive power, and chamber depth measurements into the SRK-II, SRK/T, and Barrett Universal II equations, respectively. And Barrett was used as the main formula. The obtained refractive power was as close as possible to the maximum postoperative emmetropic state. Given that implantation of the Symfony IOL results in somewhat poor near visual acuity, we used emme target for the dominant eye (when necessary to select, the IOL closest to the emmetropia was selected), and the refractive powers between the emmetropic and myopic states were used for non-dominant eye in this study which micro-monovision method was used^18^.

### Multifocal IOLs

The Tecnis ZKB00 is a diffractive bifocal IOL with an add power of + 2.75 D. This IOL contains 15 diffractive rings of the same height that dissipate light in a 50/50 ratio between the near-field focus and the far-field focus.

The Tecnis ZLB00 is a diffractive bifocal IOL with an add power of + 3.75 D with the same structure as that of ZKB00. The posterior surface of the optic components contains 18 diffractive rings.

Symfony has an echelette design with nine diffractive rings that vary in height and width. Symfony can extend the range of vision by elongating the focus. To compensate for the loss of contrast sensitivity due to the increased depth of focus, achromatic technology was used for the posterior surface of the optics to reduce the chromatic aberrations.

FineVisions a diffractive trifocal IOL that offers a combination of near vision at 3.5 D and an intermediate vision at 1.75 D. The apodization design of the diffractive rings, in which the ring height decreases toward the periphery, prevents the loss of far visual acuity at night.

### Surgical technique

One experienced surgeon (T.Y. C) performed all of the surgical procedures under topical anesthesia using a standardized suture less phacoemulsification and a 2.75 mm clear corneal incision. This is the same as the surgical method of the paper published by Yang et al. in 2018 at our institution^4^. A steep axis corneal incision was created in eyes with corneal astigmatism of more than 0.5 D. In contrast, a temporal corneal incision was made in eyes with corneal astigmatism of less than 0.5 D. The non-dominant eye was operated on first, followed by the contralateral eye one week later. Postoperatively, patients were treated with gatifloxacin eye drops (Gatiflo, Handok, Seoul, Korea) and fluorometholone 0.1% eye drops (Fumelon, Hanlim, Seoul, Korea) four times per day for 1 month.

### Patient evaluation

Our institution always strives to maintain a similar approach to evaluating patients before and after multifocal IOL surgery. Therefore, the evaluation of patients proceeded in a similar way to the paper previously published by our institution^4^. Preoperatively, all patients underwent a complete ophthalmologic examination, including corrected and uncorrected visual acuity, manifest refraction, slit-lamp bio-microscopy, and fundus examination. The patients were evaluated postoperatively on day 1, week 1, and months 1 and 3. One and 3 months after surgery, the corrected and uncorrected visual acuity, manifest refraction, defocus curve, contrast sensitivity, reading performance, and subjective satisfaction were examined.

All of the patients underwent measurement of corrected and uncorrected distance visual acuity at 5 m. The uncorrected intermediate visual acuities were measured at 60 cm and 80 cm. The uncorrected near visual acuity was measured at 33 cm, 43 cm, and 50 cm using the Snellen chart.

The defocus curves were plotted by measuring the visual acuity under the photopic condition at 5 m and adding lenses in 0.5 D increments from − 4.0 to + 2.0 D. All evaluations were done in monocular and binocular.

Contrast sensitivity was measured at 3, 6, 12, and 18 cycles per degree using a CSV-1000 chart (Vector Vision, Greenville, OH) under photopic (85 cd/m^2^), mesopic (~ 3 cd/m^2^), and mesopic with glare conditions three months postoperatively. The results were converted to log units for statistical analysis using a specific table for the CSV-1000.

The reading speed was measured 3 months after surgery at a distance of 50 cm using an iPad application (iPad Retina Display; Apple Inc., Cupertino, CA)^14^. The print size used on the reading chart ranged from 1.0 to 0 logarithm of the minimal angle of resolution (log MAR). The average reading speed in words per minute was calculated using the iPad application. Critical print size was defined as the last acuity measured before the reading speed was reduced below the 95% confidence interval of the individual’s average reading speed. The threshold print size was defined as the smallest print size that could be read and expressed in logarithm of the reading acuity determination.

The following five visual artifacts were evaluated three months after surgery: glare, halos, starburst, hazy vision, and blurred vision. To measure these artifacts, the patients were shown images and asked to rate the frequency, degree, and discomfort related to the visual artifacts as none, minimal, moderate, or severe (0, 1, 2, or 3 points for the respective levels). The mean score was calculated. In this study, artifact images and a questionnaire modified from the Quality of Vision questionnaire^19^ was used.

Patient satisfaction with near, intermediate, and far visual acuity, and spectacle dependence were also investigated using a questionnaire. Individuals rated their satisfaction with visual acuity was rated as one using the following five levels: very satisfied, satisfied, neither satisfied nor unsatisfied, unsatisfied, and very unsatisfied.

### Statistical analysis

Statistical analysis was performed using SPSS version 18.0 (SPSS, Inc., Chicago, IL). These comparisons were performed using a Bonferroni correction and a level of statistical significance set at *p* < 0.017 (i.e., 0.05 divided by 3 comparisons). Analysis of variance was used to make postoperative comparisons between the three groups.

## Supplementary information

Supplementary Information 1.

Supplementary Information 2.

Supplementary Information 3.
