# Author Correction: Comparing the visual outcome, visual quality, and satisfaction among three types of multi-focal intraocular lenses

**DOI:** 10.1038/s41598-021-88753-z

**Published:** 2021-05-03

**Authors:** Dong Won Paik, Jun Sang Park, Chan Min Yang, Dong Hui Lim, Tae‑Young Chung

**Affiliations:** 1 Department of Ophthalmology, Sungkyunkwan University School of Medicine, Samsung Medical Center, Seoul, Republic of Korea; 2 Department of Medicine, The Graduate School, Sungkyunkwan University, Seoul, Republic of Korea; 3 Nune Eye Hospital, Seoul, Republic of Korea; 4 Department of Medical Device Management and Research, Samsung Advanced Institute for Health Sciences and Technology, Sungkyunkwan University, Seoul, Republic of Korea

Correction to: *Scientific Reports* 10.1038/s41598-020-69318-y, published online 09 September 2020

The original version of the Article contained an error in the Author Information section.

“These authors contributed equally: Dong Won Paik, Jun Sang Park, Dong Hui Lim and Tae-Young Chung.”

now reads:

“These authors contributed equally: Dong Won Paik and Jun Sang Park.

These authors jointly supervised this work: Dong Hui Lim and Tae-Young Chung.”

The original Article has been corrected.

