# Supplementary figures and images for: Comparing the visual outcome, visual quality, and satisfaction among three types of multi-focal intraocular lenses
Source: Sci Rep. 2020 Sep 9;10:14832. doi: 10.1038/s41598-020-69318-y (PMC7481789; doi:10.1038/s41598-020-69318-y)

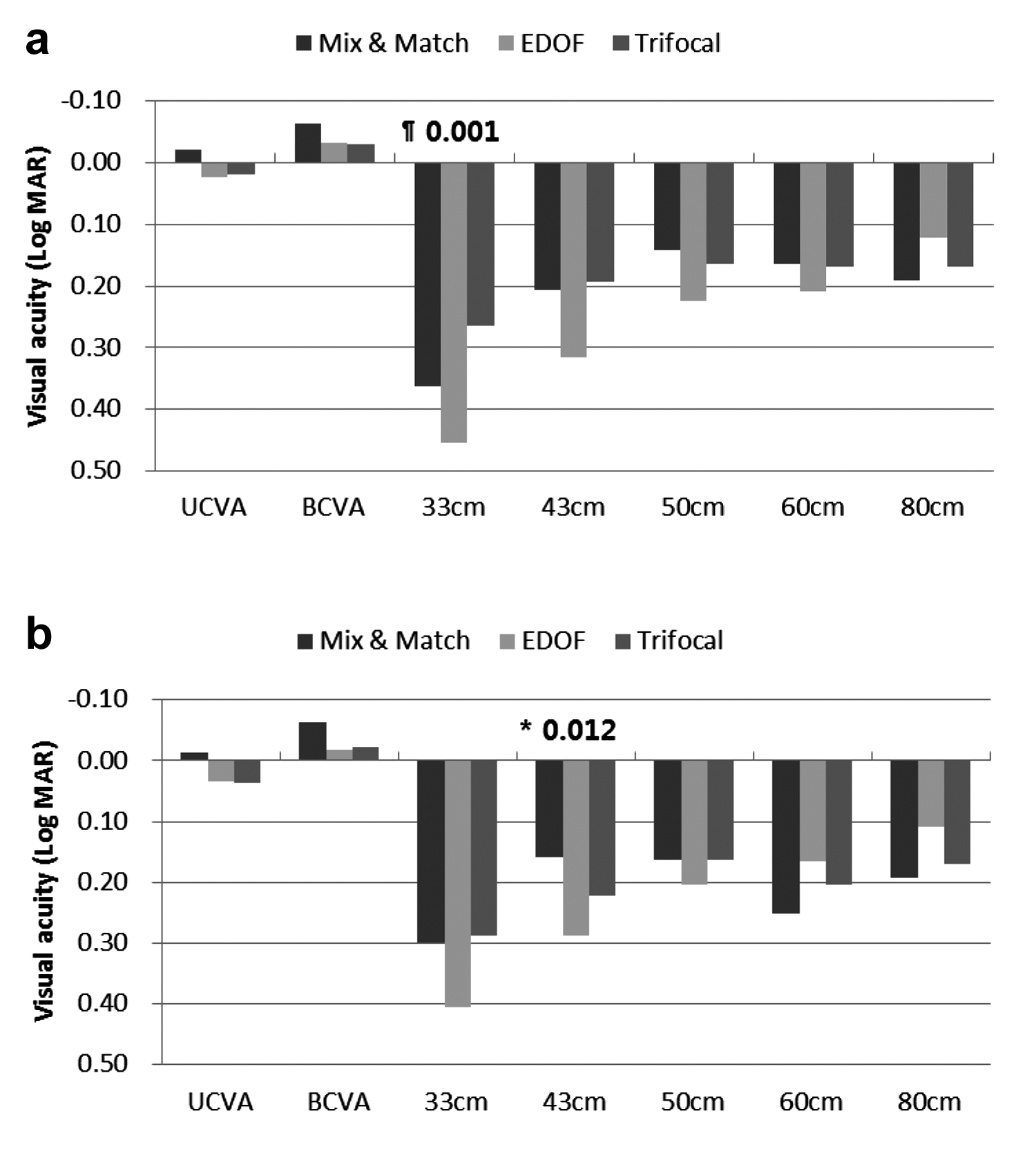

Supplement: Supplementary file 1 — Supplementary Information 1. [file 41598_2020_69318_MOESM1_ESM.tif]

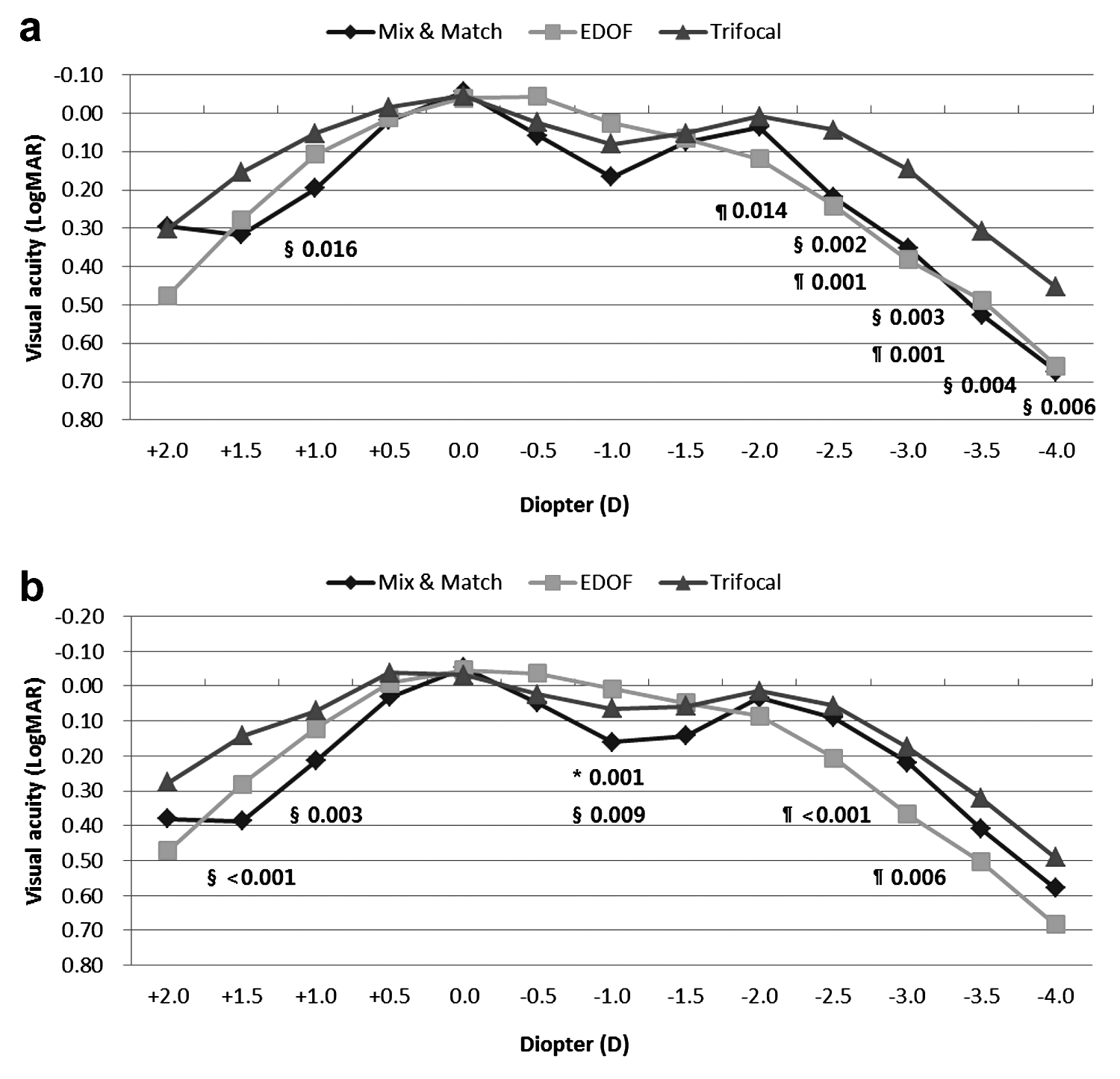

Supplement: Supplementary file 2 — Supplementary Information 2. [file 41598_2020_69318_MOESM2_ESM.tif]
