# Supplementary material for: Comparing the visual outcome, visual quality, and satisfaction among three types of multi-focal intraocular lenses
Source: Sci Rep. 2020 Sep 9;10:14832. doi: 10.1038/s41598-020-69318-y (PMC7481789; doi:10.1038/s41598-020-69318-y)
Supplement: Supplementary file 3 — Supplementary Information 3. [file 41598_2020_69318_MOESM3_ESM.pdf]

Comparing the visual outcome, visual quality, and satisfaction among three types of multi-focal intraocular lenses

Dong Won Paik, MD[1*], Jun Sang Park, MD[2,3*],

Chan Min Yang, MD[1], Dong Hui Lim, MD, PhD[1**], Tae-Young Chung, MD, PhD[1,2**]

[1]Department of Ophthalmology, Samsung Medical Center,

Sungkyunkwan University School of Medicine

[2] The Graduate School Sungkyunkwan University Department of Medicine

[3]Nune Eye Hospital

* Both authors are contributed equally to this article as first authors.
**Both authors contributed equally to this article as corresponding authors.

Representative corresponding author
Tae-Young Chung
Department of Ophthalmology, Samsung Medical Center, Sungkyunkwan University School of Medicine, #81 Irwon-ro, Gangnam-gu, Seoul 06351, South Korea
TEL: 02) 3410-3569, FAX: 02) 3410- 0074
E-mail: tychung@skku.edu

Appendix

Figure A1. Postoperative uncorrected near, intermediate, and far visual acuity

(a) Dominant eye. Subgroup analysis showed that at 33cm near distance, the EDOF group had significantly worse monocular visual acuity than the Trifocal group at three months (p=0.001). (b) Non-dominant eye. At 43cm near distance, EDOF group had significantly worse monocular visual acuity than the Mix-and-Match group at three months (p=0.012).

All outcomes were compared among the three groups. Bonferroni correction for multiple comparisons: significant p-values (p < 0.017) in bold with symbols.

*: Mix and Match vs EDOF §: Mix and Match vs Trifocal ¶: EDOF vs Trifocal

Figure A2. Defocus curves for the three groups

(a) Dominant eye. The Trifocal group had better defocus curve at near distance (-2.0 ~ -4.0D), significantly better at -2.5D (p=0.002 vs Mix-and-Match, p=0.001 vs EDOF) and -3.0D (p=0.003 vs Mix-and-Match, p=0.001 vs EDOF), respectively, at three months (p=0.001). (b) Non-dominant eye. Mix-and-Match group showed worse at -1.0D (p=0.001 vs EDOF, p=0.009 vs Trifoal), and EDOF group had significantly lower defocus curve at near distance at three months (-2.5D; p=<0.001 vs Trifocal, -3.0D; p=0.006 vs Trifocal).

All outcomes were compared among the three groups. Bonferroni correction for multiple comparisons: significant p-values (p < 0.017) in bold with symbols.

*: Mix and Match vs EDOF §: Mix and Match vs Trifocal ¶: EDOF vs Trifocal

Appendix

<Table>

Reading speed of the three groups

	Mix-and-Match	EDOF	Trifocal
Mean reading speed (wpm)	84.78±17.08	68.11±10.02	85.12±13.72
Critical print size (logRAD)	0.29±0.13	0.37±0.14	0.28±0.14
Threshold size (logRAD)	0.14±0.12	0.34±0.11	0.23±0.08

wpm: words per minute, logRAD: logarithm of the reading acuity determination
